# The Skewed Impact of Highly Cited Articles on Journal Impact Factor

**DOI:** 10.2196/45322

**Published:** 2023-09-18

**Authors:** Kazuki Ide

**Affiliations:** 1 Division of Scientific Information and Public Policy Center for Infectious Disease Education and Research (CiDER) Osaka University Osaka Japan

**Keywords:** COVID-19, journal impact factor, JIF, scientometrics, bibliometrics, infometrics, journal, assessment, research, resources, medical journal, literature, database, community, behavior

I read with interest the paper by Delardas and Giannos [1]. As the authors pointed out, journal impact factor inflation can affect the integrity of researchers as well as publishers. This phenomenon has also been reported as a “blockbuster effect” not only in the 6 high-impact medical journals reported by the authors (*Annals of Internal Medicine*, *The BMJ*, *Journal of the American Medical Association*, *The Lancet*, *Nature Medicine*, and *The New England Journal of Medicine*) but also in infectious disease journals [2]. A similar trend was observed in 200 journals with a high number of COVID-19–related publications in the Web of Science as well [3].

Delardas and Giannos [1] evaluated the impact of COVID-19–related articles on a journal-by-journal basis, but it is unknown whether they found a single article or a small number of articles that had a significant effect on impact factor. It would be helpful for readers if the authors could provide more detailed explanations.
